# From wealth to health: modelling the distribution of income per capita at the sub-national level using night-time light imagery

**DOI:** 10.1186/1476-072X-4-5

**Published:** 2005-02-10

**Authors:** Steeve Ebener, Christopher Murray, Ajay Tandon, Christopher C Elvidge

**Affiliations:** 1 Evidence and Information for Policy, World Health Organization, Av. Appia 20, 1211 Geneva 27, Switzerland; 2 Global Health Initiative, Harvard University, 104 Mt. Auburn Street, Cambridge, MA 02138, USA; 3 NOAA, National Geophysical Data Center, Office of the Director, 325 Broadway Boulder, Colorado 80303, USA

## Abstract

**Background:**

Sub-national figures providing information about the wealth of the population are useful in defining the spatial distribution of both economic activity and poverty within any given country. Furthermore, since several health indicators such as life expectancy are highly correlated with household welfare, sub-national figures allow for the estimation of the distribution of these health indicators within countries when direct measurement is difficult.

We have developed methods that utilize spatially distributed information, including night-time light imagery and population to model the distribution of income per capita, as a proxy for wealth, at the country and sub-national level to support the estimation of the distribution of correlated health indicators.

**Results:**

A first set of analysis are performed in order to propose a new global model for the prediction of income per capita at the country level. A second set of analysis is then confirming the possibility to transfer the country level approach to the sub-national level on a country by country basis before underlining the difficulties to create a global or regional models for the extrapolation of sub-national figures when no country data set exists.

**Conclusions:**

The methods described provide promising results for the extrapolation of national and sub-national income per capita figures. These results are then discussed in order to evaluate if the proposed methods could not represent an alternative approach for the generation of consistent country specific and/or global poverty maps disaggregated to some sub-national level.

## Background

Economy, income and poverty do affect and are affected by population's health in many ways.

At broad scale, the macro relationship between life expectancy and the gross national product (GNP) is well known and has been presented in different publications [1].

At a smaller scale a very robust relationship exist between an adult individual's income and that individual's health. This has been confirmed in the review done by Benzeaval and Judge [2] of sixteen studies coming from four different countries and for which the authors conclude by saying that: "All of the studies that include measures of income level find that it is significantly related to health outcomes."

The conclusion of another study performed in Tanzania [3] shows that the poorest tercile of the households in this country are the ones presenting the poorest health status indicators confirming the relationship between poverty and health status. The same study also confirms the effect of the geographic distribution of poverty on the health status of the population.

In return, high level of poverty also becomes an important factor of vulnerability for the population which becomes more exposed to diseases, especially infectious ones.

By identifying the poorest area within a country it becomes possible to plan more effective intervention aimed at improving the health status of the population and therefore potentially reducing their level of poverty. As poverty tends to be clustered in specific places it is important to have access to disaggregated data. In addition to that, aggregated, national-level poverty data tends to mask sub-national variations [4].

The development of variables that can be used as indicators of economic status is not straightforward. Even the measurement of the most basic of economic variables – such as national income levels – is fraught with problems. This reliable measurement of income is particularly problematic for low-income countries, given the lack of well-developed national income accounting methods and the large size of the "informal" sector in these economies. These problems are compounded when information is sought on the spatial and temporal changes in economic activity [5]. Recent advances in measurement and estimation techniques, though, have helped substantially. So much so that it is now routine for national statistical offices of almost all countries to report on national economic activity numbers, as well as for international organizations such as the World Bank and the International Monetary Fund to report their own versions of "adjusted" income numbers (both in local currency units as well as in purchasing-power parity terms).

However, the use of self-reported income for measurement of economic status is widely regarded to be problematic [6]. In a cross-section, income for any given household tends to be a relatively noisy indicator of its underlying longer-term economic status. From an accounting point of view, income numbers for subsistence-farming and self-employed households are particularly troublesome. In addition, respondents often perceive income-related items as being invasive and this can lead to non response bias. For these and other reasons, survey income has tended to be significantly under-reported and inconsistent with income estimated using national accounts statistics. Survey-based estimates of income are often lower than those of consumption for the same household, even though national accounts data show aggregate positive savings rates [7]. The degree of under-reporting in income has been found to vary by income deciles: lower-income households tend to be more likely to under-report than higher-income households. In several instances, poorer household have been found to report expenditure levels that far exceed reported income levels – possibly because of greater underreporting of income than of expenditure – indicating the implausible implication that the poor are chronic dissavers [8].

For all these reasons, most of the national household surveys, such as the World Bank's Living Standards Measurement Study (LSMS) [9] and many national surveys prefer to measure consumption and not income as the indicator of household welfare.

If sub-national level consumption figures are therefore available for most low income countries, through the use of these tools, the data they are producing is not comparable over countries making it impossible, for the moment, to build a consistent global map of poverty, or food insecurity, disaggregated to some sub-national level. In this context the growing uses of Geographic Information Systems (GIS) as well as the generation of new geocoded data sets might offer new perspectives in order to produce globally consistent poverty maps and help predicting the distribution of welfare at the sub-national level when reliable sub-national data are still not available. As the consumption indicators produced are not comparable over countries this attempt is done using income per capita expecting that this indicator might be comparable.

In terms of data, satellite imagery is offering great potential for global data sets depicting weather patterns plus the physical and biological environment. If most of the data sensed concerns bio-physical parameters (e.g. clouds and vegetation...) there is one parameter, sensed by some satellites that can be used in the socio-economic context: night-time light.

**Figure 1:**
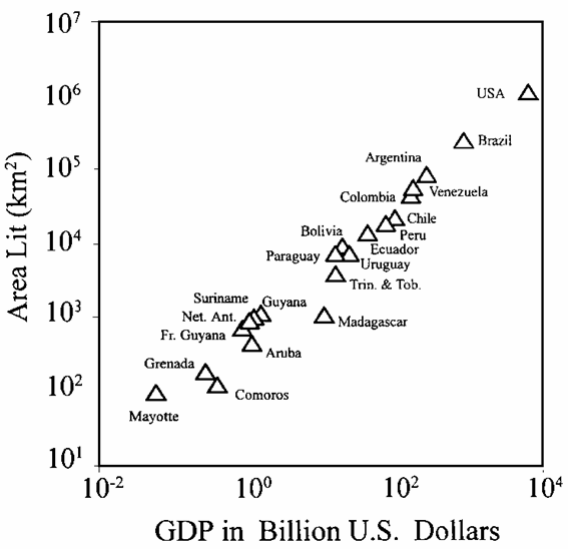
**Area lit (km2) versus 1994 Gross Domestic Product. **GDP estimates for 21 countries on a log-log plot (extracted from Elvidge et al., 12)

In their publications on the use of this parameter, Elvidge et al. [[10-12] and [13]] compares country level surface area with detected lighting at night (area lit) with population, energy usage, and economic activity. They found a strong correlation between area lit and Gross Domestic Product (GDP) for 21 countries as illustrated in Figure 1.

More recently, an attempt to obtain a global map of socio-economic parameters at the sub-national level has been made by Doll et al. [14]. In their approach, the country-level relationship found between area lit and Gross Domestic Product (GDP) was applied to a 1° × 1° resolution grid. This research showed a potential solution for obtaining sub-national distribution map for this parameter.

The methodology presented in this paper follows a different approach, using other parameters in combination with light in order to directly predict GDP per capita and adjust the results to specific conditions. It also demonstrates the role played by other environmental and socio-economic factors on this prediction.

## Results and discussion

The analysis performed at both the country and sub-national levels as well as the results obtained are presented in the coming sections.

### The country level analysis

The objective of the country level analysis is to go beyond the observations done so far [[10-13] and [14]] and to see if an other component or combination of the light information (number of cells with light, total frequency of observation, mean frequency of observation) with other parameters could provide a model for the prediction of income per capita at the country level.

The relationship between area lit and GDP shown by Elvidge et al. in 1997 [10] has already been confirmed for a larger number of countries by Doll et al. in 2000 [14] and Elvidge et al. one year later [13].

Starting from this result, a new set of analysis is performed using the different parameters described in the methods section. For the parameters stored in grids (light and surface area) the GIS tool is used in order to extract the figures based on the country delimitation.

The first set of result are reported in Table 1 and present the correlation factors existing between the different parameters expressed in log for 171 countries with:

**Table 1:** Correlation factor between the parameters used for the country level analysis

	Logdp	Logdppc	Lopopun	Losurfli	Lotofre	Lonbrpix	lomeanf
Logdppc	0.4295						
Lopopun	0.8708	-0.0700					
Losurfli	0.9340	0.3963	0.8159				
Lotofre	0.9374	0.4679	0.7808	0.9906			
Lonbrpix	0.9318	0.4249	0.7980	0.9975	0.9927		
Lomeanf	0.4413	0.5134	0.2079	0.3728	0.48	0.3709	
Losurfco	0.6723	-0.1513	0.8250	0.7442	0.6842	0.7265	-0.0164

- Logdp: log of the GDP figure (expressed in ppp)

- Logdppc: log of the GDP per capita figures (expressed in ppp)

- Lopopun: log of the UN population figures

- Losurfli: log of the area lit (km^2^)

- Lotofre: log of the total frequency of light observation

- Lonbrpix: log of the number of cells being highlighted

- Lomeanf: log of the mean frequency of light observation

- Losurfco: log of the surface area of the country (km^2^)

The logarithm of all the parameters has been used in this analysis for the following reason:

a) the relationship is expected to be linear in logs,

b) if there is heteroskedasticity, the log form is one way to remove the problem,

c) the parameters can be interpreted as elasticities if both the dependent and the independent variables are in logs

The following observation can then be extracted from Table 1:

1) Three of the light parameters are correlated to each other (lotofre, lonbrpix and losurfli). The forth one (lomeanf), not correlated to the previous ones, seems to contain a different information content connected to light,

2) Apart from the mean frequency of light observation all the parameters are more highly correlated with GDP rather than with GDP per capita,

3) There is no significant difference in the values obtained for expressing the correlation between the first three light parameters (lotofre, lonbrpix and losurfli) and GDP. Lomeanf shows a lower correlation with GDP,

4) Population presents a high correlation with GDP but none with GDP per capita. The same is observed for the surface area of the country,

5) The correlation between the first three light parameter mentioned under point 1) and population is good which is not the case with the log of the mean frequency of light observation,

6) A high correlation exists between the surface area of the country and the population.

The above-mentioned observations seem to indicate that the high correlation coefficient observed between the 3 light parameters (lotofre, lonbrpix and losurfli) and GDP is explained by the strong correlation between these parameters and population and the one between population and GDP. On the contrary the mean frequency of light observation shows a stronger correlation with GDP per capita. By predicting GDP per capita instead of GDP we therefore avoid any circularity in the model.

Based on all the observation made it is decided to consider the use of the following variable in order to model GDP per capita at the country level:

- the mean frequency of light observation as this variable present the highest correlation with GDP per capita,

- the total frequency of light observation as this parameter provides the highest correlation with GDP per capita among the other three light parameters found to be correlated to each other (lotofre, lonbrpix and losurfli). This parameter also contains more variability than the number of cells or the area lit which is an advantage when working with small areas that could be completely highlighted.

- the total population and surface area of the country. Even if these parameters do not provide a good correlation with GDP per capita they are a necessary adjustment factor for the light and population variable (density).

As the correlation between GDP per capita and the different variables may not be linear the correlation existing between the log of GDP per capita and the square of the log of these variables is analysed. This analysis shows that the square improves the prediction only for the two selected light parameters (total frequency and mean frequency of light observation) which are used as additional variables, the square of the log of population and the surface area of the country being not used.

From that point, 63 combination of the 6 variables kept for the analysis are tested in order to find a regression for modelling the log of GDP per capita at the country level. This is firstly done using the full data set, then trying to analyse the role of the climate and the one of the GDP composition by sector. Table 2 contains the information regarding the regression based on the best combination of significant variables (P > | t | < 50) using the full data set (171 countries).

**Table 2:** Information about the best regression obtained for the country level data set with F (5, 165) = 156.46, Prob > F = 0.0000, R-squared = 0.8258, Adj R-squared = 0.8205 and Root MSE = 0.20552

logdppc	Coef.	Std. Err.	t	P > |t|	[95% Conf. Interval]	
lopopun	-0.4665182	0.038419	-12.14	0.000	-0.5423734	-0.39066
lotofre2	0.0574057	0.002592	22.15	0.000	0.0522876	0.062524
lomeanfr	-2.677929	1.115337	-2.40	0.017	-4.880102	-0.47576
lomeanf2	0.9731717	0.364591	2.67	0.008	0.2533075	1.693036
losurfun	-0.1320768	0.028785	-4.59	0.000	-0.1889105	-0.07524
_cons	7.465969	0.855281	8.73	0.000	5.77263	9.154675

**Figure 2:**
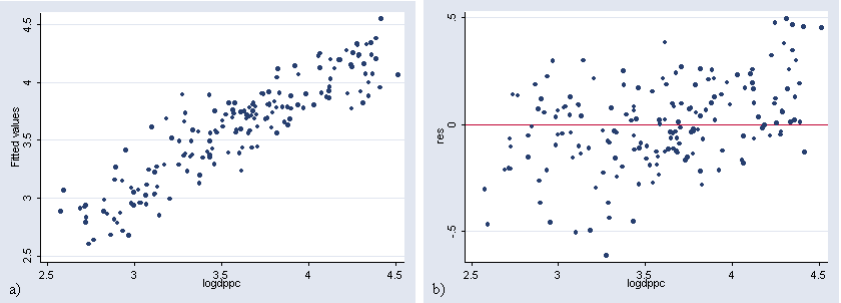
**Prediction of income per capita at the country level (in log). **a) Plot of the observed log of GDP per capita versus the predicted ones obtained with the regression in Table 2 b) Plot presenting the residuals versus the log of GDP per capita for the same regression

The plot of the observed log of GDP per capita versus the predicted figures obtained with this regression as well as the plot presenting the residuals is reported in Figure 2.

**Figure 3:**
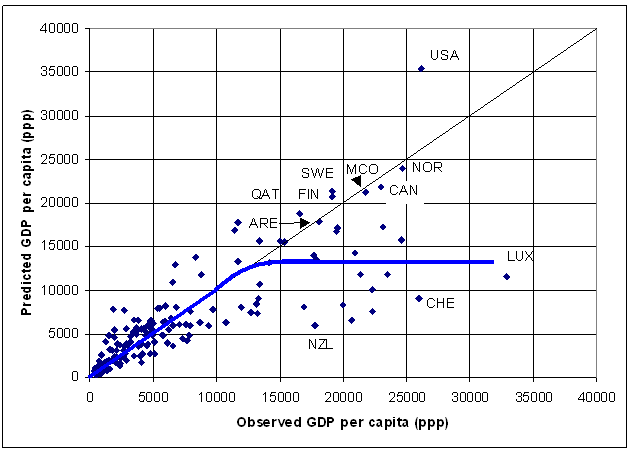
**Prediction of income per capita at the country level. **Plot report the predicted versus the observed GDP per capita for the 171 country level data set using the model reported in Table 2

The application of this model results in a significant over estimation of GDP per capita for 10 countries (United republic of Tanzania, Malawi, Zambia, Sao Tome and Principle, Tajikistan, Azerbaijan, Uzbekistan, Kyrgyzstan, Yugoslavia and Egypt) and an under estimation for most of the high income countries presenting a GDP per capita figure higher than 12'500 US$. This can be better visualized by transforming the log of GDP per capita into GDP per capita figures for the 171 countries, creating a new graph (Figure 3) similar to the one shown in Figure 2a.

Figure 3 shows that the development of the lightning infrastructure follows the economic development within a country until reaching a certain level of development after which the model is underestimating income per capita for many countries. This is explained by the fact that from this level, an increment of income is not necessarily reflected in the spatial extension of the outdoor lightning system or the creation of infrastructure that requires specific lightning at night (highways, factories, etc.).

There are however some countries for which the model gives a good estimate of GDP per capita, such as Qatar (QAT), United Arab Emirates (ARE); Finland (FIN), Sweden (SWE), Monaco (MCO), Canada (CAN), Norway (NOR). Finally, the model gives a clear overestimation of GDP per capita for the United States of America (USA).

The following explanation can be given for these countries:

- "over lighting" due to an above-average wealth of the country (e.g. for Qatar, the United Arab Emirates and maybe Monaco),

- Two explanations are possible regarding the Nordic European countries that appears in this list (Norway, Sweden and Finland) connected to the fact that the night-time light grid that is used in the context of this work is based on data collected during winter: a) specific climatic conditions during winter requiring excessive lighting of the infrastructure which may be at the origin of their location in the graph (the same observation is likely to be done for Canada). b) snow may be at the origin of an over estimation of income in these countries as snow makes lights look bigger and brighter than they appear in the no-snow condition.

- The position of the United States in the graph is related to the fact that the square of the log of the total frequency of light observation is the highest observed in the entire sample. Several hypothesis are proposed in order to explain this situation. This includes the fact that light is more easily spread due to the big habitable surface, electricity is cheap and the road network very wide.

The Root Mean Square Error (Root MSE) observed when applying this model is of 4000 US$. 27 countries are presenting an error higher than this value. The 5 countries with the highest error are: Luxembourg, Switzerland, Austria, Australia and Germany. The 5 lowest error are observed for: The Federal State of Micronesia, Burkina Faso, Haiti, Mali and Rwanda.

Trying to improve the prediction of GDP per capita at the country level by including other variables we can observe that grouping the countries according to their agricultural level is giving better results than grouping them by climatic type.

**Figure 4:**
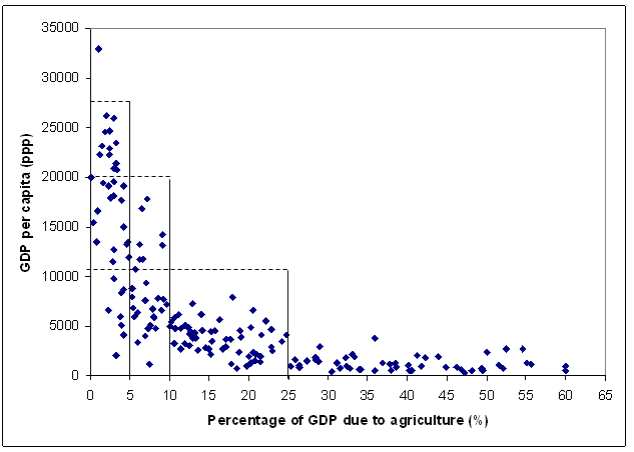
Repartition of the GDP per capita in function of the percentage of GDP due to agriculture

The figures given by the World Bank [15] or by the CIA world factbook 2001 [16] allows a decomposition of the economy into 3 sectors: agriculture, industry and services, the last two being the sectors that are the source of most of the light production. Thus the type of economy within a country has an important effect on the development of the infrastructure and then indirectly on their level of lighting. When the percentage of GPD due to the agricultural sector (Figure 4) is used to group the countries there is an improvement in the specific regression for each group.

In Figure 4, we can observe a continuous distribution of the points with breaks indicated by the vertical and horizontal lines. These breaks (5, 10 and 25 %) are therefore used for grouping the countries (170 countries used for this analysis as 1 variable is missing for 1 country part of the initial sample) and the same approach than the one described earlier is applied in order to find the regression giving the best prediction for each group as follow:

- below 5 % (38 countries): lopopun, lotofre, losurfun (Adj R-squared: 0.5785)

- between 5 and 10 % (28 countries): lopopun, lotofre, losurfun (Adj R-squared: 0.6289)

- between 10 and 25 % (55 countries): lopopun lotofre2 losurfun (Adj R-squared: 0.5463)

- above 25 % (49 countries): lopopun lotofre losurfun (Adj R-squared: 0.5512)

We can observe the same combination of variables (lopopun lotofre losurfun) in 3 of the 4 grouping but the coefficients for each of these variables are significantly different.

**Figure 5:**
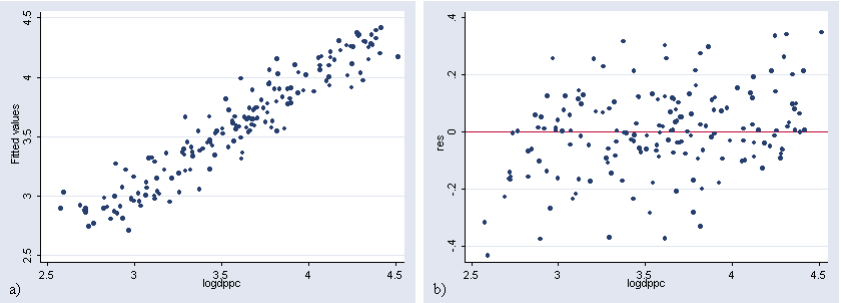
**Prediction of income per capita at the country level (grouping by agricultural level in log). **a) Plot of the observed versus the predicted log of GDP per capita figures obtained when applying the best model by agricultural level b) Plot presenting the residuals versus the log of GDP per capita for the same regression

**Figure 6:**
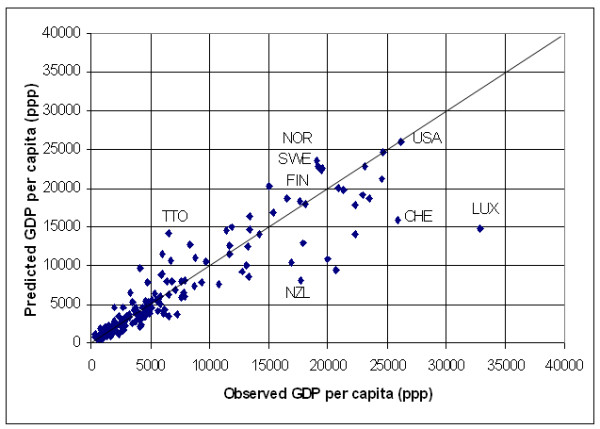
**Prediction of income per capita at the country level (grouping by agricultural level). **Plot of the predicted GDP per capita versus the observed one for the 170 countries of the data set when applying the best model found for each of the GDP agricultural contribution group.

Figure 5 shows the plot of the observed log of GDP per capita versus the predicted figures obtained as well as the plot presenting the residuals when applying these regressions to the respective group while Figure 6 shows the same graph than the one reported in Figure 5a without the logarithmic function.

From the 170 countries used in this analysis, 33 countries are presenting an error higher than the Root MSE (2877 US$). The 5 countries presenting the highest error are: Luxembourg, Australia, Switzerland, New Zealand and Singapore. The 5 lowest error are observed for: Chad, Somalia, Bangladesh, Iran and the Republic of Moldova.

### The sub-national level analysis

The sub-national level analysis has two objectives: to examine the possibility of applying the country level approach to the sub-national level on a country-by-country basis and to explore the possibility of generating global or regional models to be used for countries where sub-national data are either missing or are deemed to be unreliable.

This analysis is based on the log of the same parameters successfully used at the country level. The population, total frequency and mean frequency of light observation as well as the surface area figures are extracted from the grids described in the methods section using the boundaries of corresponding sub-national administrative or statistical units.

In order to keep the consistency with the values used for country level analysis, the sub-national figures for the population and the total frequency of light observation are adjusted by applying an homogeneous factor and rounding the resulting figures to the closest integer number.

#### Transferring the country level approach to the sub-national level

The analysis of the correlation factor existing at the sub-national level between each variables and GDP per capita (expressed in log) shows significant heterogeneity from one country to another. For few countries, some variables even appear as being not significant for the prediction of GDP per capita at the sub-national level. The list of variables observed in the country specific regression giving the best prediction vary therefore also from one country to another. In this case we do not look for models that were based on significant variables but take the one that is presenting the highest Adj R-squared value as the number of observation is generally low. The Adj R-squared obtained for these regressions varies from 0.2492 for The Netherlands to 1.00 for Portugal.

This analysis indicates that it may be difficult to find a universal global or regional model that could be applied at the sub-national level. It is nevertheless important to underline the fact that at least one of the light variables is present in all the regression which is not the case for the population or the surface area of the sub-national units.

For 3 countries (Italy, the Republic of Korea and the United Kingdom) the high correlation existing between GDP and population seems to indicate that these sub-national figures have been generated using a linear model based on population only. This finding demonstrates that not all reported sub-national data are reliable and emphasizes the need for independent methods for generating sub-national estimates. The sub-national level data for these 3 countries are therefore taken out from the sample for all the analysis reported in this publication.

**Figure 7:**
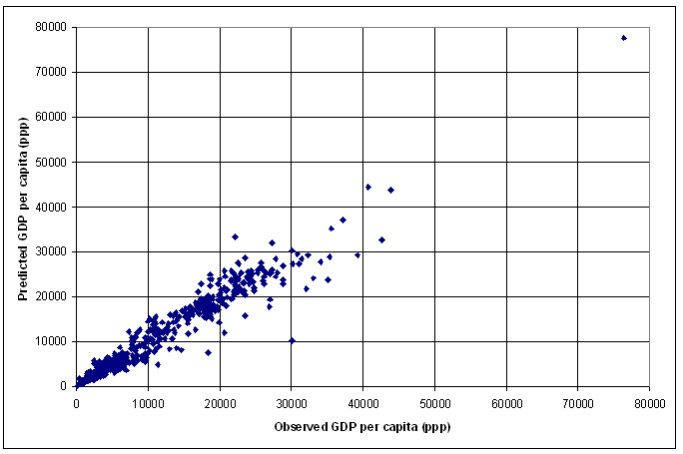
**Prediction of income per capita at the sub-national level (country specific model). **Plot of the predicted GDP per capita versus the observed one when applying the sub-national country specific model.

Figure 7 summaries, in a unique graph, the result obtained when applying the country specific regression and converting the results into GDP per capita figures.

Table 3 list the number of units with an error bigger than the country specific Root MSE (also listed) as well as the percentage of units this correspond to. This represents a total of 97 sub-national units (mean value of 12.8 % of unit per country). All the countries presenting a national GDP per capita higher than 15,000 US$ as well as the 3 Latin American countries part of the sample (Argentina, Brazil and Mexico) are at the top of this list. The countries for which the prediction obtained is bellow the Root MSE for all the units are: Bangladesh, Greece, India, Mozambique, Portugal and South Africa.

**Table 3:** Root MSE, number of units outside the 1*RMSE range and percentage of the total number of unit this represents when applying the country specific model

Country	Root MSE	Number of unit outside 1*RMSE	% of the number of units
DEU	4615	17	48
USA	3546	24	47
ARG	3240	7	29
ESP	2312	4	25
BEL	2384	2	18
BRA	1405	5	18
SVN	1427	2	16
MEX	1777	5	15
IDN	1651	4	14
RUS	2693	11	14
AUT	1405	1	11
THA	2074	9	11
FRA	1605	2	9
NLD	2669	1	8
FIN	1437	1	5
SWE	1264	1	4
CHN	981	1	3

Among these 97 units, 31 are containing at least one city with a population larger than 1,000,000 inhabitants (including some capital cities). Their presence in the list can be explained by the high concentration of buildings which represents vertical highlighted structures for which the satellite sensor is not able to capture the total intensity of the light being produced. This also confirms that in some countries the capital cities are more highly lit than their in country counterparts. Five other units contains oil and/or gas production infrastructure (2 in Indonesia, 2 in the USA and 1 in the Russian Federation). The separation of the lights produced by gas flares from the city lights could be the explanation for the under prediction of income per capita in these units. The difficulty in these cases is to know if the income produced by this activity remains within the concerned unit or goes directly to the government or even outside the country. This observation is to be extended to the offshore infrastructures that should also be considered in the model.

Even if no particular characteristics are identified for the remaining 61 units, these results illustrate the possibility to transfer the approach developed at the country level to the sub-national level on a country-by-country basis. This analysis also demonstrate the need to have income figures for some sub-national units in order to find the regression that provides the extrapolated figures for the remaining ones. This concerns units containing big cities (more than 1,000,000 inhabitants) and units containing oil and/or gas production. Additional analysis have to be performed in order to make sure that these are the only cases or if other specificity also have to be taken into account.

An out of sample analysis should also be performed in order to determine what is the smallest sample (% of all the units) necessary to obtain a regression providing prediction of acceptable quality.

#### Generating a global or regional model for the prediction of sub-national figures

As the availability of disaggregated income data is very poor, and despite the observation made in the previous section regarding the heterogeneity of the country specific sub-national model, tests and analysis are done in order to see if it is possible to generate a model that would allow the generation of sub-national income per capita series for countries where no sub-national figures are available. Four approaches are used:

- application of the country level model described in Table 2

- generation of a model based on all the sub-national data (with and without country dummy)

- generation of a model grouping the countries by climatic types

- generation of a model grouping the countries by agricultural level using the GDP composition by sector

The best results being obtained with the last approach, only this one is described in details in this section. For the other approaches only the major findings are reported.

Applying the country level regression to the sub-national data set and presenting the results on a summary graph (all the countries on the same graph) gives an acceptable prediction for sub-national units presenting a GDP per capita figure bellow 5,000 US$. Above this limit the application of this approach under estimate GDP per capita. When we generate country specific graphs we can observe that the important variety of income per capita figures at disposal in the sample is in fact at the origin of the observation done earlier on the summary graph and not the result of a good prediction for each country considered in the sample. This emphasizes the need to make country specific graph for analysing the results.

This analysis also demonstrates that applying the country level regression to the sub-national level may introduce an important quantitative error. In this regards, the attempt done by Doll et al. in their publication [14] should only be considered as a qualitative result.

When using the entire sub-national sample for generating one unique regression, the combination of variables giving the best results (Adj R-squared = 0.7692) is the same set found for the country level model (Table 2). The coefficients observed for each of the variables are also very close to the ones observed for the country level regression (-0.4955732*Lopopun + 0.059074*lotofre2 - 2.959219*lomeanfr + 1.176556*lomeanf2 - 0.1686316*losurfun + 7.774052). These small differences have an impact on the prediction of the income per capita figures for the units presenting a value higher than 10,000 US$ (ppp). The sub-national model gives better result than the country level model in only a few countries: Austria, Brazil, Greece, Portugal, Spain, South Africa, Thailand and the Netherlands.

If we integrate a dummy variable in the same regression we can observe one more time the same combination of variables than the one obtained for the country level model (Table 2) but the coefficients are slightly different. This alternate approach improves the prediction of the sub-national GDP per capita figures (Adj R-squared = 0.8971) reducing the dispersion of the estimates obtained for the middle and high income countries.

**Figure 8:**
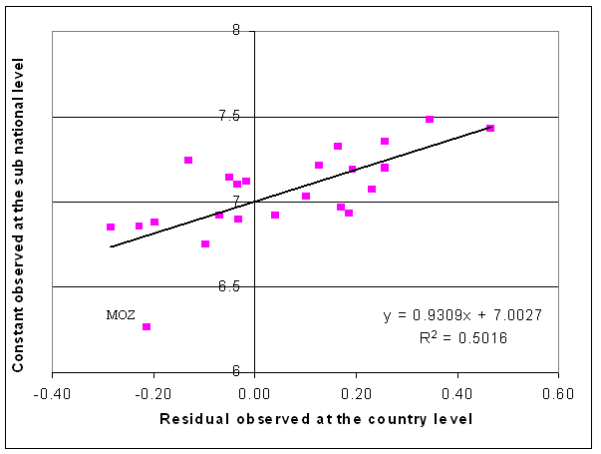
**Correlation between the country and the sub-national level prediction. **Illustration of the correlation existing between the residual observed at the country level and the country specific constant observed at the sub-national level

Another advantage of this approach is the fact that a good correlation exist between the country specific residual observed when applying the regression in Table 2 at the country level and the country specific constant obtained when applying the sub-national level model including the dummy variable (Figure 8).

In theory we could then apply the regression mentioned in Figure 8 on the residual found for a particular country during the country level analysis in order to find the constant to be used for predicting the sub-national GDP per capita figure for the same country. Even if the Adj R-squared for this regression is quite high (0.50) we can observed that Mozambique already represents an outliner indicating that using this correlation may unfortunately also generate important errors in the estimation of the country specific constant.

In conclusion, even if this approach is giving better results than the previous ones (application of the country level regression and generation of a unique regression without country dummy) and is presenting an important advantage due to the existing correlation with the country level model we can not consider that the results obtained are of sufficient quality for applying it to other countries where we would not have any sub-national figures.

Grouping the countries part of the sub-national data set by climatic types improves the prediction of GDP per capita for Bangladesh, France, India, The Netherlands, The Russian Federation, Sweden and Spain. This improvement mainly concerns sub-national units presenting an observed GDP per capita higher than 10,000 US$ and is not related to a particular climatic type.

When the sub-national sample is grouped according to the percentage of GDP associated with agriculture in each country, using the same cut off point than for the country level analysis (Figure 4), the following set of countries are obtained:

- Below 5 % of GDP due to agriculture: Austria, Belgium, Finland, France, Germany, Portugal, Slovenia, Spain, Sweden, the Netherlands and USA,

- Between 5 and 10 %: Argentina, Brazil, Greece, Mexico, Russian Federation and South Africa,

- Between 10 and 25 %: China, Indonesia, and Thailand,

- More than 25 %: Bangladesh, India and Mozambique.

**Figure 9:**
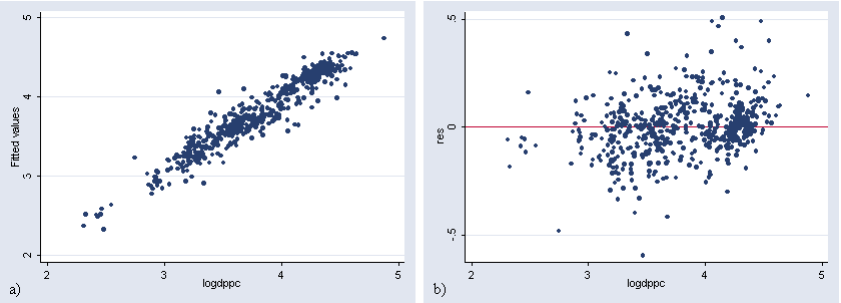
**Prediction of income per capita at the sub-national level (grouping by agricultural level in log). **a) Plot of the observed versus the predicted log of GDP per capita obtained when applying the regression giving the best prediction with the sub-national data set separated into groups based on the percentage of GDP due to agriculture and with a country specific dummy variable b) Plot presenting the residuals versus the log of GDP per capita for the same regression

**Figure 10:**
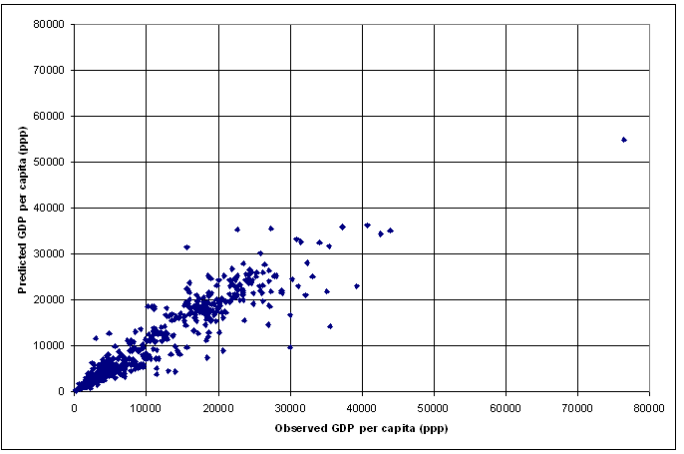
**Prediction of income per capita at the sub-national level (grouping by agricultural level). **Plot of the predicted versus the observed GDP per capita for the sub-national data set when applying the models grouping the countries using the percentage of GDP due to agriculture including a country dummy.

The regression found for each group (based on significant variables except for the country specific constant and the country dummy) are presenting an Adj R-squared varying from 0.5731, for the countries presenting a percentage of GDP due to agriculture between 5 and 10 %, to 0.8013 for the countries with a percentage higher than 25 %. Like for the grouping by climatic type, the list of variables found is different for each group. Figure 9 shows the plot of the observed versus the predicted log of GDP per capita as well as the plot presenting the residuals obtained when applying these regressions. Figure 10 contains the same graph than the Figure 9a when taking out the logarithmic function.

Using this approach, 108 sub-national units are presenting a prediction error bigger than the Root MSE, which represent a mean percentage of units by country of 21 %. The countries for which this grouping induces a significant improvement of the prediction, compare to the models used previously, are: Bangladesh, India, Belgium, Spain, France, the Netherlands, Portugal, Slovenia, Sweden, USA, Finland and the Russian Federation which represents 5 more countries than when using the grouping by climatic types. We can also observe that this improvement does mainly concerns countries were agriculture represent less than 5 % of the country GDP. Only exceptions: Bangladesh, India and the Russian Federation.

Even so, 3 of the countries for which the unique regression with dummy variable was not capturing the variability of GDP per capita before (Mexico, Argentina and Greece) are still presenting the same type of results.

Due to the small number of observation for each grouping it is not possible to identify if a correlation exists between the residuals observed at the country level and the country specific sub-national constant making it difficult to generate a regional model for these groupings using the same approach than the one described previously (Figure 8).

## Conclusions

This study demonstrates that night-time lights data are useful in generating estimates of both national and sub-national GDP per capita figures. Because night-time lights are produced with zero reliance on national reporting data, they provide an independent measure of economic activity.

The country level results reported here confirm the conclusion given by Doll et al. [14] regarding the possibility of expanding the relationship between area lit and GDP to a larger number of countries. But these results raise the question whether the high correlation observed between these two parameters is in fact not coming from the good correlation existing between population and area lit and the one between population and GDP.

By introducing parameters other than area lit in the regression we have demonstrated the possibility of independent estimation of GDP per capita at the country level with a high level of confidence. This offers an interesting possibility for completing country level data sets for which data are missing or are in error. Special attention should nevertheless be paid to small densely populated territories with high level of lighting (e.g. Singapore and Monaco). In these areas, the models tend to underestimate GDP per capita as light produced by vertical infrastructures like high rise buildings fails to expand the area of lighting. This effect may be reduced if brightness information on the lights is available.

The graph reported in Figure 3 demonstrate that satellite observed area lit and percent frequency of lighting can be successfully used for the prediction of GDP per capita at the country level until a certain limit of economic development above which the relationship breaks down. GDP per capita estimates can be improved by developing models for groups of countries having similar climate or having similar proportions of the country GDP associated with agriculture. Even if the Root MSE observed when using these two models are close to each other our preference goes to the model grouping the countries by agricultural level as it produces fewer outliers. The disadvantage of adding more sub-groupings than the one proposed is that the number of countries in each group becomes small, reducing the strength of the model.

**Figure 11:**
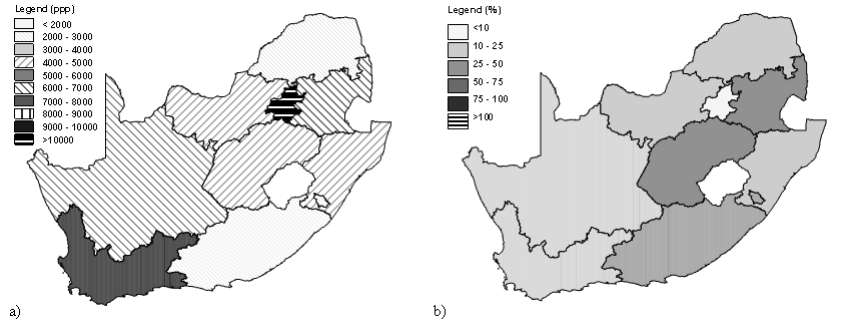
**South Africa, application of the country specific model. **a) Distribution of the predicted GPD per capita figure when applying the country specific model b) Distribution of the prediction error when applying the same model

The result demonstrate that the approach developed for the country level can also be apply at the sub-national level but only through the generation of country specific models. Figure 11 illustrate the result obtained by the application of the country specific model found for South Africa as well as the distribution of the prediction error expressed in percentages.

In this regard the possibility to generate country specific model for the extrapolation of sub-national income per capita figure is offering an interesting solution for countries where sub-national data on welfare are not available or where the application of other methods (e.g. small area estimation) would be difficult.

Nevertheless, the fact that income per capita figures are necessary for some sub-national units in order to generate the country specific model represent a limitation to the application of this approach. If the analysis done in the context of this work already gives an indication of the type of units for which it would be necessary to have a good estimation of the income per capita figures additional work would be necessary in order to confirm this list, maybe add other type of units and also have a better indication regarding the minimum number of units for which input data are needed to insure a good prediction. In addition to that, further analysis are also needed in order to define the level of desegregation to which it would be possible to go based on the developed approach. It will finally be necessary to consider including the amount of light due to gas or oil production infrastructure in the model if the income they are producing stays within the sub-national units where they are located.

Despite the caveats, the results obtained clearly demonstrate an interesting potential for making independent estimates of GDP per capita at the sub-national level especially for low income countries where the prediction obtained are of good quality and the need for them definitively the most important. Improving the data sets required to operationalize this approach may be far easier than improving the national reporting of sub-national economic data.

It would nevertheless be important to compare the results obtained by this approach with other methods also producing sub national estimated using consumption indicators in the context of poverty mapping exercises [[4] and [17]]. In addition to that, the possibility to maybe apply this approach to consumption indicators instead of income per capita figure should be explored as this would then represent an additional method, presenting the advantage of being less sophisticated, for the generation of desegregated country specific poverty maps. An other advantage would be that this approach would then not only be applicable to data collected in the context of the World Bank Living Standards Measurement Study (LSMS) [9] but also to the ones collected for example in the context of the WHO World Health Survey [18]. Even if this new instrument is presenting an additional advantage, by also collecting health indicators at the household level, work has to be done in order to confirm that the data collected are sub-nationally representative.

**Figure 12:**
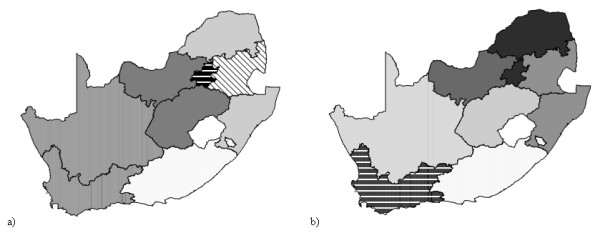
**South Africa, application of the model grouping the countries by agricultural level. **a) Distribution of the predicted GPD per capita figure when applying the model grouping the countries by agricultural level. b) Distribution of the prediction error when applying the same model (see Figure 11 for the legend).

Trying to generate a consistent global or regional poverty map desegregated to some sub-national level, the grouping of the sample at disposal by climate or percentage of GPD due to agricultural level is definitively improving the prediction but does not provide us with a model generating consistent estimates for all the countries. Between these two grouping the preference again goes to the second one which improves the estimation for the middle and high income countries. The result obtained when applying this model to South Africa as well as the distribution of the prediction error expressed in percentages are reported in Figure 12 as an example. Despite being the best regional model analysed in the context of this research the grouping by agricultural level is still producing significant error when applying it on a country by country basis (Figure 12b) compare to the country specific model itself (Figure 11b).

In this regards, the analysis performed is not offering us the possibility to generate consistent global map showing the sub-national distribution of income per capita figures.

These type of exercises are sorely needed to improve our knowledge regarding the health and well being of people in the poorest areas of the world. In this context night-time lights remain a useful data set for the evaluation of the impact of international efforts to improve the economic and therefore health conditions of these populations. It would therefore be important to pursue the type of work described in this paper and to see if the results obtained could not be improved.

For both the country and the sub-national level model, the combination of the two groupings used in the context of this present work could for example improve the proposed models but this would have to be confirmed through additional analysis.

The more recent global night-time light mosaic that NOAA has compiled is another element which might improve the results presented in the context of this work. This new global mosaic has the advantage of covering a longer period of observation (full years instead of the six month composite used in this study) which would improve the homogeneity of the distribution of the number of observation per pixel and also reduce the effect of the snow observed on images collected during winter. These new products were processed with major improvements in the exclusion of all but the highest quality segments of the individual orbits. They also include one information that was missing in the data set used for the context of this work: the digital number brightness of the lights. It has been found that adding in brightness of the lighting greatly improves the relationship to variables such as electric power consumption and GDP. Even if some difficulties to make products with brightness values remains, due to saturation in urban centers and the lack of on-board calibration, this new generation of grids could allow a significant improvement of the models described in the present paper. Another advantage of this data set is that the NGDC is producing a full global composite for each year from 1992 through 2004 allowing therefore for trend analysis.

Before being able to test such new source of spatially distributed information it would be important to correctly address the question of the lack of documentation regarding the methods used for generating sub-national income estimates reported by individual countries in case this indicator would provide better results than consumption when trying to generate a consistent global poverty map. Inclusion of erroneous data may lead to misleading interpretations (see the case of the data for the United Kingdom, Italy and the Republic of Korea in the sub-national level analysis section).

Such discrepancies also underline the value of high quality geospatial data for use in making independent estimates of economic activity. This for example includes standardization of the basic vector GIS layers (national and sub national borders for example) in order to insure a proper use of the information collected at the sub national level or stored in different raster layers (night-time light and population for the present work). Among the different initiatives that are trying to answer the need for standardization in this area we can mention the Second Administrative Level Boundaries data set project (SALB). The first objective of this project is to create a redistributable Second Administrative Level Boundaries global data set (SALB) representative of January 2000 to be used with the GIS technology. The information finally collected has extended the period of representativity of the database for finally covering the period 1990-present. A process has also been put in place in order to insure the updates of the database in the future. You can find all the relevant information about this project as well as the data already available on the project web site [19]. The growing use of this database and more specifically of its specific coding scheme should improve the availability and comparability of sub national income figures in the future.

Finally, the shifts observed between all these layers of geographical distributed information is emphasizing the need for defining a "master" that could be used as a ground reference when generating or working with them. If the precision of its georeferencing is confirmed, the global mosaic of satellite images that are now publicly available (e.g the Landsat mosaic) could for example constitute this master.

## Methods

In order to perform the analysis at the country and at the sub-national level it was necessary to compile existing data sets on income as well as geographically distributed parameters that would be used to model these figures using a GIS. These concerns:

- night-time light imagery

- population

- international and sub-national boundaries

- surface area

- climate

The data sets used for the context of this paper are presented now. In order to insure the consistency of the analysis, all the data sets compiled or created were adjusted to 1995, which is the year of representativity of the only night-time light grid available at the beginning of this research.

We utilized GDP per capita data that have been collected at both the country and sub-national level. For the country level, GDP in International Dollars (GDP I$) for 171 countries have been calculated for the year 1996 from GDP figures expressed in local currency unit using price level data [20] and were adjusted to be representative of 1995.

For the sub-national level, GDP figures representative of the first or second administrative or statistical level have been collected for 26 countries (653 units) from the 5 continents (Table 4). These figures were homogenised in order to obtain a final data set expressed in Power Purchasing Parity (PPP) representative of 1995. A country specific adjustment factor has been applied to these figures in order to keep the consistency with the country figures before dividing them by their corresponding population.

**Table 4:** Source of the income figures for the sub-national analysis

**Country**	**ISO 3 Code**	**Admin-Stat./Level/Nb unit**	**Representativity**	**Source of the data**	**Income level**
Argentina	ARG	Adm/1/24	1995	National statistics agency, Argentina	Middle

Austria	AUT	Adm/1/9	1994	Sozialstatistic Austria	High

Bangladesh	BGD	Admin/2/19	1993	Statistical Yearbook of Bangladesh, 1994	Low

Belgium	BEL	Admin/2/11	1994	MaconUSA	High

Brazil	BRA	Admin/1/27	1997	IBGE; Brazil	Middle

China	CHN	Admin/1/30	1998	Statistical Yearbook of China, 1998	Low

Finland	FIN	Stat/3/19	1997	Statistics Finland	High

France	FRA	Stat/2/22	1994	MaconUSA	High

Germany	DEU	Stat/2/35	1994	MaconUSA	High

Greece	GRC	Admin/1/13	1994	Statistical Office of Greece	High

India	IND	Admin/1/25	1991	Statistical Outline of India 1999–2000	Low

Indonesia	IDN	Admin/1/27	1994	Statistical Information Services, Indonesia	Middle

Italy	ITA	Stat/2/20	1994	MaconUSA	High

Mexico	MEX	Admin/1/32	1995	INEGI, Mexico	Middle

Mozambique	MOZ	Admin/1/10	1997	Instituto Nacional de estatistica, Mozambique	Low

Netherlands	NLD	Stat/2/12	1994	MaconUSA	High

Portugal	PRT	Stat/2/7	1994	MaconUSA	High

Russian Federation	RUS	Admin/1/77	1996	State Com. Of the Russian Federation on Statistics	Middle

Slovenia	SVN	Stat/2/12	1996	Statistics Slovenia	Middle

South Africa	ZAF	Admin/1/9	1994	Statistics South Africa	Middle

Republic of Korea	KOR	Admin/1/14	1995	National Statistical Office of Korea	High

Spain	ESP	Stat/2/16	1994	MaconUSA	High

Sweden	SWE	Stat/3/21	1996	Statistics Sweden	High

Thailand	THA	Admin/1/76	1995	Chulalongkorn University, Bangkok	Middle

United Kingdom	GBR	Stat/2/35	1994	MaconUSA	High

United States of America	USA	Admin/1/51	1997	Harvard University, USA	High

The night-time light grid used has been provided by NOAA's National Geophysical Data Center (NGDC). This grid data set is the result of a 6-month 1 km resolution composite based on images collected between October 1994 and March 1995 by the U.S. Air Force Defence Meteorological Satellite Program (DMSP) Operational Linescan System (OLS) [10]. Only the grid with the distribution of lights associated with human settlements has been used in the context of the present research. Due to improvements in the algorithms used during the processing, this grid is different than the one used by Doll et al. for their publication [14]. By comparing these two grids it has been possible to identify some of these differences and to take advantage of their respective specificity for generating the grid used here. From this grid it possible to extract 4 parameters connected to light observation at night which are capturing a different information making it possible to extract the figures at the country or sub-national level for the analysis:

- the number of cells highlighted at night

- the area lit (surface area being highlighted at night) which is giving an indication of the extension of the highlighted surfaces,

- the total frequency of light observation (obtained by adding the percents frequency of light detection observed in each cell on a given surface) which is giving an indication of the total intensity of the highlighting

- finally, the mean frequency of light observation, in the highlighted areas, which gives us an indication of the dispersion of this intensity. For example: one pixel with a 100 % of light observation or 100 pixels with 1 % of light observation are giving the same value for the total frequency of light observation but a totally different figure for the mean frequency of light observation and area lit.

These information being stored in a grid it is possible to extract them at the country or sub-national level for the analysis.

Regarding population, the country level data that have been used are the UN population figures for the year 1995 [21]. For the sub-national level the Gridded Population of the World (GPW) version 2 has been selected [22] as being consistent with the UN country level data set. In addition to that, this data set is offering the possibility to use GIS in order to make spatial analysis at the sub-national level.

Climate is known to have an important influence on many elements on the earth surface, including human behaviour and well being [23], and also on the need for specific lighting of infrastructure. The Köppen climate classification distribution grid derived by the Food and Agriculture Organization (FAO) from the International Institute for Applied Systems Analysis (IIASA) data sets has therefore been used for the context of this project [24]. Using this grid it is possible to determine the general climate of any geographical entity (national or sub-national). Köppen categories are based on the annual and monthly averages of temperatures and precipitation.

Five major climatic types are recognized in this system, each type being designated by a capital letter (each of them being divided into sub types):

A – Tropical moist climates: all months have an average temperature above 18 degrees celsius.

B – Dry climates: with deficient precipitation during most of the year

C – Moist mid-latitude climates with mild winters

D – Moist mid-latitude climates with cold winters

E – Polar climates: with extremely cold winters and summers

The delimitation of the units of analysis (countries and sub-national units), corresponding to the GDP figures collected for the context of this study, have also been prepared in a format that could be used in the GIS tool. In order to insure the consistency from one country to another the delimitation of the international borders has been adjusted to 1995.

Finally, the surface area of the units of analysis has also been included in the data set.

Two types of software have been used in analysing national and sub-national income: a Geographic Information System (GIS) and a statistical package. In order to take advantage of the specificity of the two modes of representation observed in the compiled data set (vector and raster) we have worked with the ArcView GIS software (version 3.2) for the vector part complemented by the Spatial Analyst extension (version 1.1) for the raster part. As no statistical analysis could be done directly in the GIS software the statistical support was provided by the STATA software (Version 5).
